# Multimodality Imaging of a Giant Aortic Valve Papillary Fibroelastoma

**DOI:** 10.1155/2013/705101

**Published:** 2013-07-28

**Authors:** Nowell M. Fine, David A. Foley, Jerome F. Breen, Joseph J. Maleszewski

**Affiliations:** ^1^Division of Cardiovascular Diseases, Department of Medicine, Mayo Clinic, 200 First Street SW, Rochester, MN 55905, USA; ^2^Department of Radiology, Mayo Clinic, 200 First Street SW, Rochester, MN 55905, USA; ^3^Division of Anatomic Pathology, Department of Laboratory Medicine and Pathology, Mayo Clinic, 200 First Street SW, Rochester, MN 55905, USA

## Abstract

Papillary fibroelastomas (PFEs) are benign cardiac tumors arising from endocardium. They are commonly found on valvular surfaces and average 1.0–1.5 cm in size. Though often asymptomatic, PFEs can lead to potentially severe complications, primarily due to their embolic potential. Surgical resection is recommended for all symptomatic or large PFEs. We report the case of a patient presenting with cardiovascular symptoms who was found to have a very large aortic valve PFE, as diagnosed by histopathologic examination following surgical resection. Multimodality cardiovascular imaging demonstrates the classic morphologic findings, including a pedunculated appearance and oscillating “frond-like” surface projections.

## 1. Introduction

Papillary fibroelastomas (PFEs) are said to be the second most common benign primary cardiac tumor (behind myxomas) and account for at least 75% of all valvular tumors [1]. Grossly, these tumors typically have a pedunculated appearance with multiple surface projections, causing them to look like a sea anemone. Histologically, they are composed of numerous avascular, endocardium-lined fronds [2, 3]. Though benign and often asymptomatic, PFEs can lead to potentially severe complications, including embolic phenomena (either from the mass itself or adherent thrombus) such as transient ischemic attack or stroke, pulmonary embolism, or obstruction of coronary arteries causing angina or even myocardial infarction [4]. Previously identified predominantly at autopsy, PFEs are now more often discovered by echocardiography (transthoracic or transesophageal) for evaluation of symptoms or incidentally [2]. 

## 2. Case

**Figure 1:**
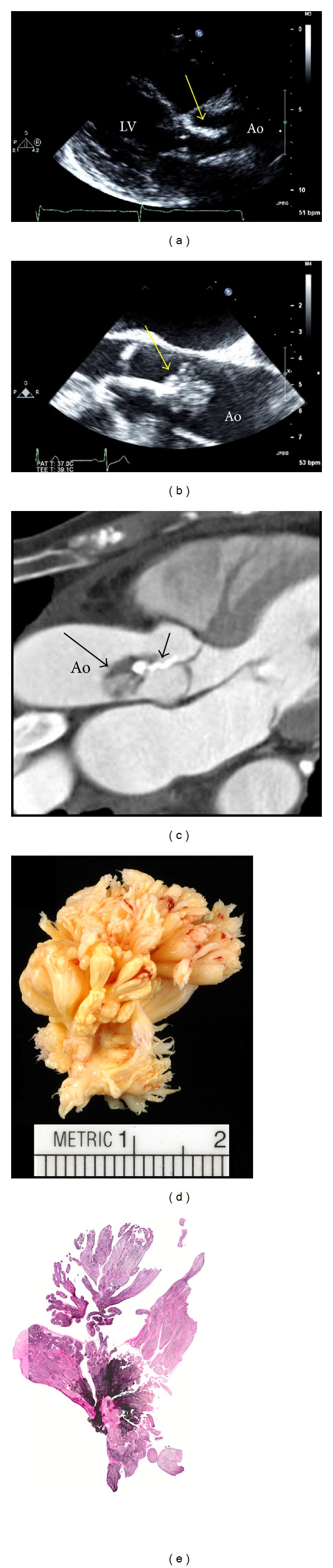
Transthoracic (a) and transesophageal echocardiography (b) imaging of a large pedunculated aortic valve mass (yellow arrows) subsequently identified as a papillary fibroelastoma (PFE). Cardiac-gated CT angiography (c) demonstrates the numerous papillary fronds (long arrow) emanating from the heavily calcified central stalk (short arrow). Following surgical resection, the mass was confirmed as a PFE by gross and histologic inspection (d) including Verhoeff-Van Gieson staining (e). Ao: aorta, LV: left ventricle.

An 84-year-old woman with a history of hypertension and coronary artery disease (CAD) was referred for echocardiography evaluation of a systolic murmur. She complained of mild exertional dyspnea and intermittent chest discomfort. Routine investigations, including electrocardiogram, chest X-ray, and laboratory testing were noncontributory. Transthoracic echocardiography was suspicious for a mass attached to the aortic valve extending into the aorta (Figure 1(a)). Transesophageal echocardiography confirmed a large pedunculated mass measuring 4.2 cm in length attached to the surface of the aortic valve right coronary cusp, with oscillating projections on its surface (Figure 1(b), see Supplementary Material available online at http://dx.doi.org/10.1155/2013/705101). The mass was causing mild outflow obstruction by Doppler-flow interrogation and mild aortic valve regurgitation. Surgical resection was recommended due to the embolic potential of the mass. Due to the patient's history of CAD, preoperative cardiac-gated computed tomography (CT) angiography was performed in favor of invasive coronary angiography due to the risk of catheter disruption of the mass causing an embolic event. While significant CAD was excluded, the images illustrated the heterogeneous nature of the mass, including the heavily calcified stalk and emanating surface projections, with minimal calcification of the aortic valve cusps (Figure 1(c)). The mass was successfully resected, sparing the aortic valve. Gross pathologic inspection (Figure 1(d)) was consistent with a large PFE, confirmed by histologic examination with Verhoeff-Van Gieson staining demonstrating numerous avascular, endocardium-lined fronds (Figure 1(e)). 

## 3. Discussion

Cardiac PFEs can arise on any endocardium-lined structure and average 1.0–1.5 cm in size at the time of diagnosis [1, 2]. While their etiology is unknown, most regard them to be either reactive or truly neoplastic (hamartomatous or otherwise). PFEs most often occur on cardiac valvular surfaces, and over 95% occur in the left heart [1]. The most commonly involved valve is the aortic valve. PFEs arising on semilunar valves occur with roughly equal frequencies on the ventricular and arterial surfaces of the valve, while those occurring on atrioventricular valves have a predilection for the atrial surface. Nonvalvular PFEs represent approximately 15% of all cases and have been reported arising on the endocardium of both the left and right ventricles (particularly the septum), atria (including the septum, appendages, Eustachian valve, and Chiari network), papillary muscles, and even the intimal surface of coronary artery ostia. PFEs usually occur in isolation; however, multiple synchronous tumors are not uncommon and have been reported as lining the ventricular cavity with a “carpet-like” appearance [5]. 

There are no clear risk factors for developing PFEs. It has been speculated that they develop due to a reaction of the endocardium to injury, and they have been observed to occur with increased frequency in patients with chronic rheumatic heart disease, hypertrophic cardiomyopathy, and prior surgery or mantle field radiation. Lambl's excrescences are degenerative strands that are also thought to be caused by endocardial injury. Although the fronds of Lambl's excrescences appear similar microscopically to those of PFEs, controversy remains as to whether they represent two distinct entities or a spectrum of the same pathologic process. Adherence of multiple adjacent Lambl's excrescences into larger and more complex forms has been described as “giant Lambl's excrescences” [6]. Unlike PFEs, Lambl's excrescences rarely occur on the arterial surface of semilunar valves, and PFEs are larger and have a more pedunculated morphology [7]. 

Although they can occur at any stage of life, PFEs are frequently discovered between the 4th and 8th decades. The diagnosis is usually made by 2-dimensional echocardiography. TTE is useful for evaluating suspicious masses; however, TEE is often required for those that are more complex appearing or poorly visualized. Echocardiography demonstrates a mobile pedunculated mass with a speckled or shimmering appearance and a stippled pattern at the edges. PFEs can often be identified using cardiac CT or magnetic resonance imaging (MRI). In addition to their morphologic characteristics, the tumors have a reported CT attenuation of 52 to 69 Hounsfield units, while typical cardiac MRI features include intermediate signal on T1 and intermediate-to-high signal on T2-weighted sequences, no suppression with fat saturation, with enhancement following gadolinium contrast injection [8]. The majority of PFEs are readily identifiable by echocardiography alone in the absence of technically difficult images, and the use of cardiac CT in our report was particular to this case. 

Although benign and slow growing, PFEs carry a risk of embolic complications. The majority of PFEs are found incidentally at the time of echocardiography or other cardiac imaging, cardiac surgery, or autopsy [2]. The most common clinical presentation is due to embolic events. PFEs are firmly attached to the endocardial surface making dislodgement unlikely; however, emboli can form either from fragments of the fragile frond-like papillary tissue or from shallow surface thrombus. In addition to cerebral and cardiac emboli, PFEs have been reported to cause mesenteric, renal, and limb ischemia. PFEs have also been known to cause interference with valvular function, resulting in either obstruction, regurgitation, or a combination. The patient reported in this case presented with symptoms of dyspnea and intermittent chest discomfort. PFEs causing angina have been well described and are most common with aortic valve PFEs, due to either coronary emboli from the tumor or obstruction of coronary ostia [8]. Symptoms typically resolve following tumor resection, as occurred in this case. 

Surgical resection is recommended for all symptomatic PFEs, or those ≥1 cm in size. The management of smaller asymptomatic PFEs is controversial, although some advocate surgical resection for any PFE, regardless of the presentation. Due to their pedunculated structure, they can usually be readily resected, sparing the underlying structure. Surgical resection is generally well tolerated and curative.

 Although this case represents one of the largest PFEs to be imaged, PFEs of similar size and even larger have been previously reported [1]. The size of this tumor allowed for excellent TEE delineation of some of the classical features of PFEs, especially the mobile frond-like projections on its surface. The PFE described in this report is exceptional not only in size but also in its heterogenous composition, and the large calcified segment of the stalk is an unusual feature. Despite this, the tumor was resected without difficulty or disruption of aortic valve function.

## Supplementary Material

Transesophageal echocardiogram of the aortic valve and proximal ascending aorta in long axis, demonstrating a large papillary fibroelastoma attached to the aortic valve.
